# The role of pediatricians and pediatric researchers in the climate crisis

**DOI:** 10.1038/s41390-026-04760-8

**Published:** 2026-01-17

**Authors:** Marie-Louise Herrmann, Cynthia F. Bearer, Jasper V. Been, Benjamin W. Ackermann

**Affiliations:** 1 Division of Neonatology, Department of Women’s and Children’s Medicine, University Hospital Leipzig, Leipzig, Germany; 2 Department of Child and Adolescent Psychiatry, University Medical Center Hamburg-Eppendorf, Hamburg, Germany; 3 Division of Neonatology, UH Rainbow & Babies Hospitals, Department of Pediatrics, Case Western Reserve University, Cleveland, OH USA; 4 Division of Neonatology, Department of Neonatal and Paediatric Intensive Care, Erasmus MC Sophia Children’s Hospital, University Medical Centre Rotterdam, Rotterdam, Netherlands; 5 Department of Obstetrics and Gynaecology, Erasmus MC Sophia Children’s Hospital, University Medical Centre Rotterdam, Rotterdam, Netherlands

## Abstract

- Call for action to implement special interest groups regarding planetary health within pediatric research societies
- Pointing out the role and responsibility of pediatricians and pediatric researchers in times of climate crisis
- Raising awareness about research gaps, e.g., regarding mitigation strategies
- Focusing on the carbon footprint of conferences, hospitals and, laboratories
- Recommendations for sustainable medical and research practice

## Children at the center of the climate crisis

**Fig. 1:**
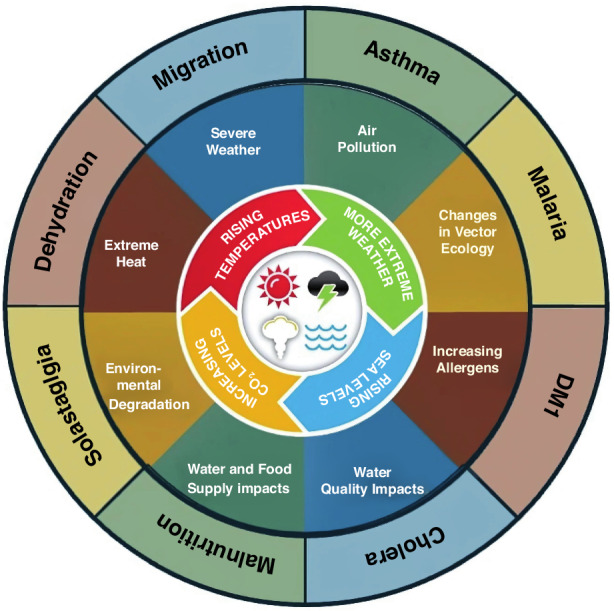
Visualization of the “vicious cycle” of the climate crisis (center circle), including tipping points (global warming, rising sea levels, increasing atmospheric carbon, and more extreme weather events). The middle circle shows resulting environmental changes (e.g., air pollution). In the outer circle, we give examples of associated effects on child health (e.g., asthma, malnutrition, solastalgia (i.e., eco-anxiety)). (Modified from^11^ by adding diseases relevant to pediatrics in the outer ring based on.^1^).

Many children born today will be alive at the predicted peak of the climate crisis in approximately 2100. The increasing rate of extreme weather events (e.g., heatwaves, floods, wildfires, hurricanes, droughts) is associated with global warming. Importantly, the climate crisis disproportionally affects poorer countries and vulnerable populations, even though they contribute far less to global emissions. Children and adolescents will bear the highest burden of climate change over their lifetime,^1^ as shown in Fig. 1. In recent years, these impacts have increased. The intensification of these risks has made the existential issue of “climate crisis and health” a focus of politics and society. Climate change has also found its way into the consciousness of many healthcare professionals. The prominence of the climate crisis stands in stark contrast to the relative paucity of pediatric research aimed at assessing and addressing its impact on perinatal and child health. Also, children are not considered a specific risk group in the climate adaptation policies of many countries.^2^

## Health impacts and the role of pediatric care

Alarmingly, many children deal with anxieties about the climate every day. About 75% of youths and young adults have expressed these concerns in surveys on the climate crisis.^3^ When asked how worried they are about the climate crisis, a higher percentage of participants in countries more threatened by climate catastrophes answered “extremely or very worried” (the Philippines at 84%, India at 68%), as compared to countries less threatened (Finland at 44%, USA at 46%).^3^ About 80% feel that older generations have failed to protect our planet.^3^

Pediatric patients and pediatric professionals are personally experiencing the initial effects of the climate crisis, as described in Fig. 1.^4,5^ As pediatric healthcare professionals and researchers, we must take responsibility for our patients’ somatic, psychological, and holistic future. These areaswill have lifelong cumulative effects that require further investigation.

## Research gaps and the need for action

Research on planetary health and the climate crisis is becoming increasingly important, resulting in a new field of research. New projects, studies, and reviews focused on children and adolescents have emerged recently: Pubmed shows 333 references for 2019-2024 vs. 75 articles for 2003-2018 (search terms: climate change and child health). A deeper understanding of the impact of the climate crisis on child health is as essential as effective mitigation strategies(i.e., strategies designed to reduce causes of the climate crisis) and adaptation strategies (i.e., those focused on reducing the consequences of climate change, including those relevant to child health). Politicians depend on sound scientific findings to make informed short-, medium-, and long-term decisions. Many good ideas concerning climate change mitigation and adaptation exist, but there is little reliable data on effective strategies relevant to child health. In addition, specific clinical conditions and crises require different strategies: for example, combating increasing malnutrition is undoubtedly different from preventing heatstroke in toddlers. In 2023, 4,080 articles were published on adaptation and mitigation in the context of the climate crisis, but only 17% of these dealt with the health aspects.^4^

As pediatrichealthcare professionals, we are also responsible for educating caregivers about the impact of the climate crisis on their children’s health. Knowledge about increasing health risks due to the climate crisis, such as severe dehydration in heat waves, must be made available to parents prophylactically. This is especially important if children already have relevant pre-existing conditions (e.g., heart failure requiring diuretics). This knowledge transfer should not only take place in acute situations, but as part of routine examinations and developmental check-ups. It should be carried out in a similar way to other medical counseling, e.g., recommendations for safe sleep, accident prevention, etc., and should be structured in national and international guidelines for routine healthcare visits in childhood and adolescence. Pediatricians and pediatric researchers must therefore fulfill a dual role: (1) collecting and analyzing data and thus creating new knowledge while (2) passing this knowledge on to our colleagues, patients, and policymakers.

## Greening pediatric healthcare and research

We must implement massive changes to achieve the international goal of climate neutrality. Worldwide, the healthcare sector contributes approximately 4.6% to global carbon dioxide emissions, with the national share in Germany estimated at 5.8% and the share in the US at approximately 8.5%.^4^ We need to monitor the current carbon footprint and implement carbon reduction strategies in the healthcare system. Our expertise is needed to identify specific areas of potential reduction (i.e., ‘hotspots’) within pediatrics and develop appropriate reduction concepts. Benchmark and implementation studies allow us to measure the success of our reduction ideas and projects. Networks within and between pediatric societies are needed to foster the exchange of ideas and research results.

Successful local hospital projects reducing the ecological footprint of the healthcare system need to be shared with the international research community, including those of the adult world. Hospitals and laboratories could thus become green (reduce their carbon footprint) faster and more efficiently, and intellectual resources could be conserved and used for new projects. Initiatives such as reducing the footprint of hospital laboratories, both clinical and research, should be promoted within the clinical and research communities.^6^

Interest groups within research societies enable us to share knowledge and ideas, promote new projects, and make use of established networks. Also, conferences/congresses need to become sustainable (water fountains instead of plastic bottles, supporting vegetarian/vegan meals (meat production is responsible for large carbon emissions^7^), encouraging sustainable transport conditions^8^), and should include the climate crisis as an essential topic for sessions and workshops. Conference/congress speakers can enrich their presentations with aspects associated with planetary health by including the topic within their presentation.^9^

To put this into practice, we founded the Special Interest Group “Planetary Health” within the Epidemiology, Public Health, and Health Services Research Section of the European Society of Pediatric Research in 2023.^10^ We aim to: (1) support and coordinate research on planetary child health, especially in the context of the climate crisis, (2) initiate a cross-border exchange of experiences,and (3) be an example for other research organizations.

Many international and national organizations, including pediatric associations and health authorities, are also beginning to include planetary health and climate resilience in their agendas. These joint efforts will be crucial in developing a coordinated global strategy to protect children’s health in the age of climate change.
